# Increased ABCC4 Expression Induced by ERRα Leads to Docetaxel Resistance via Efflux of Docetaxel in Prostate Cancer

**DOI:** 10.3389/fonc.2020.01474

**Published:** 2020-08-28

**Authors:** Houbao Huang, Jing Li, Jing Shen, Ling Lin, Xu Wu, Shixin Xiang, Yawei Li, Yujie Xu, Qijie Zhao, Yueshui Zhao, Parham Jabbarzadeh Kaboli, Mingxing Li, Xiang Li, Weiping Wang, Qinglian Wen, Zhangang Xiao

**Affiliations:** ^1^Department of Urology, Yijishan Affiliated Hospital, Wannan Medical College, Wuhu, China; ^2^Department of Oncology and Hematology, Hospital (T.C.M) Affiliated to Southwest Medical University, Luzhou, China; ^3^Laboratory of Molecular Pharmacology, Department of Pharmacology, School of Pharmacy, Southwest Medical University, Luzhou, China; ^4^South Sichuan Institute of Translational Medicine, Luzhou, China; ^5^Department of Pharmacy, Yijishan Affiliated Hospital, Wannan Medical College, Wuhu, China; ^6^Department of Oncology, The Affiliated Hospital of Southwest Medical University, Luzhou, China

**Keywords:** prostate cancer, docetaxel resistance, ERRα, ABCC4, bioinformatics

## Abstract

Docetaxel is a major treatment for advanced prostate cancer (PCa); however, its resistance compromises clinical effectiveness. Estrogen receptor-related receptor alpha (ERRα) belongs to an orphan nuclear receptor superfamily and was recently found to be closely involved in cancer. In the present study, we found that ERRα was involved in docetaxel resistance in PCa. Overexpression of ERRα conferred docetaxel resistance in PCa cell lines, and cells with ERRα downregulation were more sensitive to docetaxel. Among the drug resistance-related genes, ABCC4 demonstrated synchronous expression after ERRα manipulation in cells. Moreover, both ERRα and ABCC4 were overexpressed in the docetaxel-resistant cell, which could be reversed by ERRα knockdown. The knockdown of ERRα also reversed the reduced drug accumulation in the docetaxel-resistant cell. We also demonstrated for the first time that ABCC4 was a direct target of ERRα as determined by the CHIP and luciferase assays. Bioinformatics analysis revealed high expression of ERRα and ABCC4 in PCa patients, and a number of potential ERRα/ABCC4 targets were predicted. In conclusion, our study demonstrated a critical role for ERRα in docetaxel resistance by directly targeting ABCC4 and stressed the importance of ERRα as a potential therapeutic target for drug-resistant PCa.

## Introduction

Prostate cancer (PCa) is one of the most common and heritable malignancies and the third leading cause of cancer death in men, especially in the western population (1). Docetaxel provides effective control for most of PCa, including high-risk or localized advanced PCa, metastatic hormone-sensitive PCa, metastatic castrate-sensitive, and castration-resistant PCa (2–5). However, docetaxel chemotherapy and docetaxel-based combination therapy (docetaxel with androgen deprivation therapy) also have certain limitations and problems clinically. Docetaxel can moderately prolong the overall survival (OS) of patients but gradually lead to disease progression on account of inherent or acquired docetaxel resistance (6, 7). In addition, a combination treatment of docetaxel and other medicines (like abiraterone, Enzalutamide) even can give rise to clinical cross-resistance (8, 9). Multiple mechanisms, including reduced intracellular concentrations of drug (increased drug efflux), androgen-receptor (AR) activation, central transcription factor, changes in B-tubulin isotype expression, apoptotic pathways, angiogenesis, mutations in tumor suppressor proteins, and so on, have been demonstrated to be involved in the development of docetaxel resistance (7, 10, 11). Docetaxel resistance is one of the major hurdles that must be overcome in order to achieve successful treatment of PCa with chemotherapy and docetaxel-based combination therapy.

Estrogen receptor-related receptor alpha (ERRα) is one of the three subtypes of ERRs (ERRα/NR3B1, ERRβ/NR3B2, ERRγ/NR3B3) (12) and belongs to an orphan nuclear receptor super family of DNA-binding transcription factors. Increasing scientific evidence has indicated that ERRα is closely involved in carcinogenesis and tumor progression, including breast cancer (13), prostate cancer (14), and bone tumor progression which are related to advanced prostate cancer (15). Indeed, in PCa, ERRα is detected in cancer cell lines, xenografts, and cancerous lesions (16), and it is regarded as a negative prognostic predictor (17). However, thorough knowledge about the functional role of ERRα in PCa is lacking.

The protein encoded by ABCC4 is an important member of the ATP-binding cassette (ABC) membrane transporter family which can transport various molecules across extra/intra cellular membranes, and is involved in the export of endogenous signaling molecules and chemotherapeutic agents (18, 19) and the transport of steroid hormones (20). The inhibition of ABCC4 has been shown to be beneficial for atherothrombotic disease due to its involvement in vascular biology and in platelet functions (21–23). Importantly, the broad-spectrum resistance in cancer cells is often caused by the overexpression of ABC transporters (24), and upregulation of ABCC4 transcription is correlated with multidrug resistance in various kinds of cancer (25, 26). Hence, ABCC4 is also known as the multidrug resistance protein 4 (MRP4). In PCa, higher-level expression of ABCC4 is reported in malignant prostate tissues when compared to benign prostate tissues (27). Importantly, ABCC4 is considered as a key determinant of docetaxel resistance in PCa cells because of reversed drug resistance by inhibiting ABCC4 expression (28).

A growing body of scientific evidence has demonstrated that both ERRα and ABCC4 are highly expressed in PCa and relate to tumor progression. It is also notable that ABCC4 is crucial for multi-drug resistance, especially docetaxel resistance. This study aimed at discovering whether ERRα could regulate drug resistance-related genes and revealing the relationship between ERRα and ABCC4.

## Materials and Methods

### Cell Culture and Reagents

Human epithelial PCa cells (PC3) were cultured in F-12K medium with 10% fetal bovine serum, and C4-2B was cultured in 4:1 ratio of DMEM and F12 medium with 11% fetal bovine serum and 1.1% T-Media. To establish docetaxel-resistant cells including PC3/DR and C4-2B/DR, we used the increasing concentration method. Firstly, we confirmed the IC_50_ of PC3 and C4-2B for docetaxel, respectively. We used the IC_50_ as a starting concentration. Cells were cultured in increasing concentrations of docetaxel for about 2 months and were subsequently stimulated with docetaxel once in a while. Docetaxel was purchased from Sigma (USA). The antibodies used were ERRa (ab76228, Abcam) for Western blots, ERRa (PA5-28390, Thermo Fisher) for ChIP, Rabbit monoclonal for MRP4 (ab233382, Abcam), and beta-actin (ab115777, Abcam) and HRP-conjugated secondary antibodies (A0208, Beyotime).

### MTT Assay

Cell viability was measured by the 3-(4,5-Dimethylthiazol-2-yl)-2, 5-diphenyltetrazolium bromide (MTT) assay as previously described (29).

### Total RNA and Protein Extraction

Total RNA was isolated from the PCa cell by Trizol (Invitrogen) in accordance with the instructions. Total protein was lysed from the PCa cell in RIPA buffer with complete protease inhibitor and phosphatase inhibitor. Both RNA and protein were stored at −80°C.

### Plasmid Construction

Full-length human ERRα cDNA was synthetized and inserted into plenti-puro vector using homologous recombination. The plasmid was named plenti-ERRα (Genscript Co. Ltd., Nan Jing, China). Cells were transfected with plenti-ERRα using Lipofectamine 2000 (Thermo Fisher, USA), and a stable clone was established by puromycin selection. The sh-ERRα sequence which was well-identified before was synthetized and inserted into a pLKO.1-TRC vector by homologous recombination. The knockdown plasmid was named pLKO-shERRα (Genscript Co. Ltd., Nan Jing, China). Lentiviral shRNA production, infection, and stable cell selection were performed as previously described (30).

### Quantitative Real-Time PCR Analysis

Reverse transcription was done using the FastKing RT kit (with gDNase). Real-time qPCR was performed by using FastFire qPCR PreMix. Both RT and qPCR kits were purchased from TIANGEN (China). Primers for qPCR were as follows: ERRα Forward: CCACTATGGTGTGGCATCCTGT, Reverse: GGTGATCTCACACTCGTTGGAG; ABCC1 Forward: CCGTGTACTCCAACGCTGACAT, Reverse: ATGCTGTGCGTGACCAAGATCC; ABCC2 Forward: GCCAACTTGTGGCTGTGATAGG, Reverse: ATCCAGGACTGCTGTGGGACAT; ABCC3 Forward: GAGGAGAAAGCAGCCATTGGCA, Reverse: TCCAATGGCAGCCGCACTTTGA; ABCC4 Forward: CTGTTGGAGGATGGTGATCTGAC, Reverse: CTGCTAACTTCCGCATCTACTGC; ABCC5 Forward: GGCTGTATTACGGAAAGAGGCAC, Reverse: TCTTCTGTGAACCACTGGTTTCC; ABCG2 For-ward: GTTCTCAGCAGCTCTTCGGCTT, Reverse: TCCTCCAGACACACCACGGATA; MDR1 Forward: GCTGTCAAGGAAGCCAATGCCT, Reverse: TGCAATGGCGATCCTCTGCTTC; β-actin Forward: CACCATTGGCAATGAGCGGTTC, Reverse: AGGTCTTTGCGGATGTCCACGT.

### Western Blot

Electrophoretic analysis of different proteins was carried out on SDS–PAGE and transferred to a PVDF membrane. Experiment was performed following the standard laboratory protocol as previously reported (29).

### Chromatin Immunoprecipitation Assay (ChIP)

The ChIP assay was performed as described previously (29). Cross-linked chromatin was incubated overnight with anti-ERRα. The precipitated DNA was analyzed by qPCR. Primers used were as follows: ABCC4 P1 Forward: TTACCCGGCTTTCTTGAGGT, Reverse: GGTTTGGGAAGACTGGGAGA; ABCC4 P2 Forward: GGGTGGATATGAAGAGCAGC, Reverse: TCTAAGCATGGCCTGTCTCC; ABCC4 P3 Forward: GGTGACAGAGCAAGACCCTA, Reverse: ACTCTTGTCTTAGGGTCTTGTCA.

### Luciferase Reporter Assay

The wild-type or ERRα-binding site-deleted ABCC4 promoter region was subcloned into the pMIR–REPORT reporter (Life Technologies). ERRα binding sites P1 and P2 were deleted separately (Genscript Co. Ltd., Nan Jing, China). The luciferase activity was determined as previously described (31).

### High Performance Liquid Chromatography (HPLC)

Chromatographic separation was performed on an Agilent 1,290 LC system using a ZORBAX Eclipse Plus C18 column (2.1 × 100 mm, 1.7 μm, Agilent Technologies, USA). The column temperature was set at 35°C, and the flow rate was 0.30 mL/min. The temperature of the autosampler was maintained at 4°C. The mobile phases consisted of A (Acetonitrile) and B (Distilled water with 0.1% formic acid). The gradient elution program was as follows: 0–1.5 min, A, 10–30%; 1.5–5 min, A, 30–95%; 5–7 min, A, 95%; and return to the initial condition for a 3-min equilibration. The injection volume was 20 μL. The UV absorbance was monitored at 227 nm.

### Accumulation of Docetaxel in C4-2B/DR Cells

All the cells were seeded into 24-well plates and allowed to grow overnight. To study the intracellular accumulation of docetaxel, the cells were incubated with docetaxel in complete culture medium at 37°C for 30 min. Afterwards, the cells were washed twice with ice-cold PBS. The intracellular docetaxel was determined by HPLC after cell lysis. The values were normalized to the protein content.

### cBioPortal Database Analysis

The cBioPortal for Cancer Genomics (http://www.cbioportal.org/) is one of the major and authoritative sources of cancer genomics data which is crucial for our research. Relevant information about ERRα and ABCC4 in PCa including mRNA expression levels, association with clinical parameters, genetic alteration, protein expression, interaction network, and co-expression genes was analyzed by cBioPortal.

### Statistical Analysis

Statistical analysis was performed using GraphPad Prism 7. All data are expressed as mean ± SD from three separate experiments performed in triplicate. The co-expression level was analyzed by Spearman's correlation test, and a cutoff of 0.3 was used. The Pearson test and Spearman rank test were used for correlation analysis. The Kaplan-Meier survival curve by using the log-rank test was used for overall survival analysis. *p* < 0.5 was considered statistically significant.

## Results

### The Influence of ERRα Manipulation on Docetaxel Sensitivity

**Figure 1:**
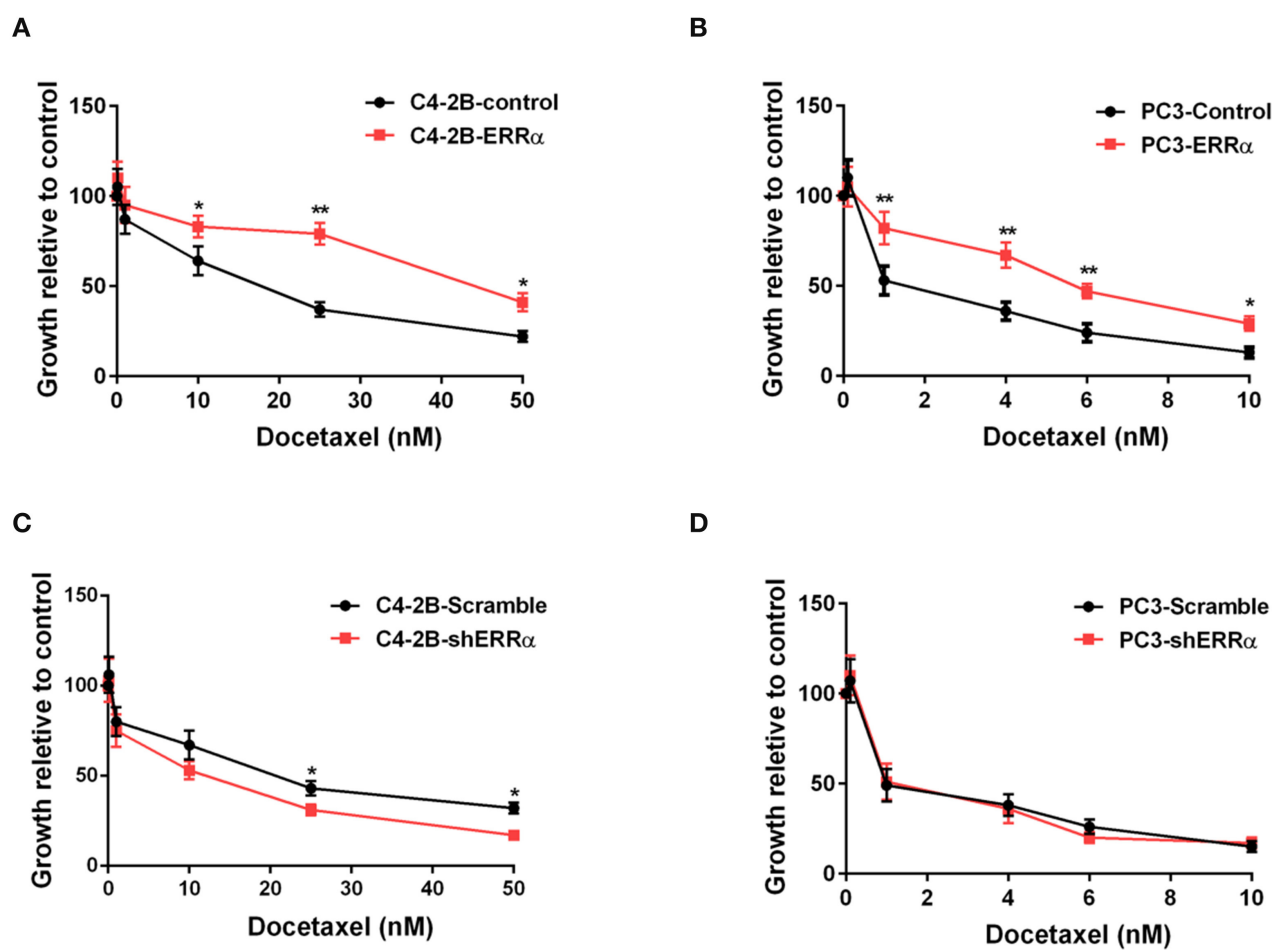
Effects of ERRα overexpression and knockdown on docetaxel sensitivity. **(A,B)** Effect of ERRα overexpression on docetaxel sensitivity in C4-2B and PC3 cells was determined by MTT assay at 24 h. **(C,D)** Effect of ERRα knockdown on docetaxel sensitivity in C4-2B and PC3 cells was determined by MTT assay at 24 h. *p < 0.05, **p < 0.01 compared with control.

In order to assess the influence of ERRα expression on docetaxel sensitivity in PCa, we chose two PCa cells (C4-2B and PC3) treated with different concentrations of docetaxel (ranging from 10 to 50 nM) following the overexpression and knockdown of ERRα. Results demonstrated that C4-2B and PC3 cells with elevated expression of ERRα displayed significant resistance to docetaxel treatment when compared with the control group (Figures 1A,B). At the same time, C4-2B cells with ERRα knockdown were more sensitive to docetaxel (Figure 1C). However, there is no significant difference in PC3 cells with or without ERRα knockdown to docetaxel treatment (Figure 1D). We also confirmed the result with the colony formation assay (Supplementary Figure 1). The above data suggested that ERRα up-expression was linked to docetaxel resistance in PCa cell lines.

### The Influence of ERRα Manipulation on Drug Resistance-Related Genes

**Figure 2:**
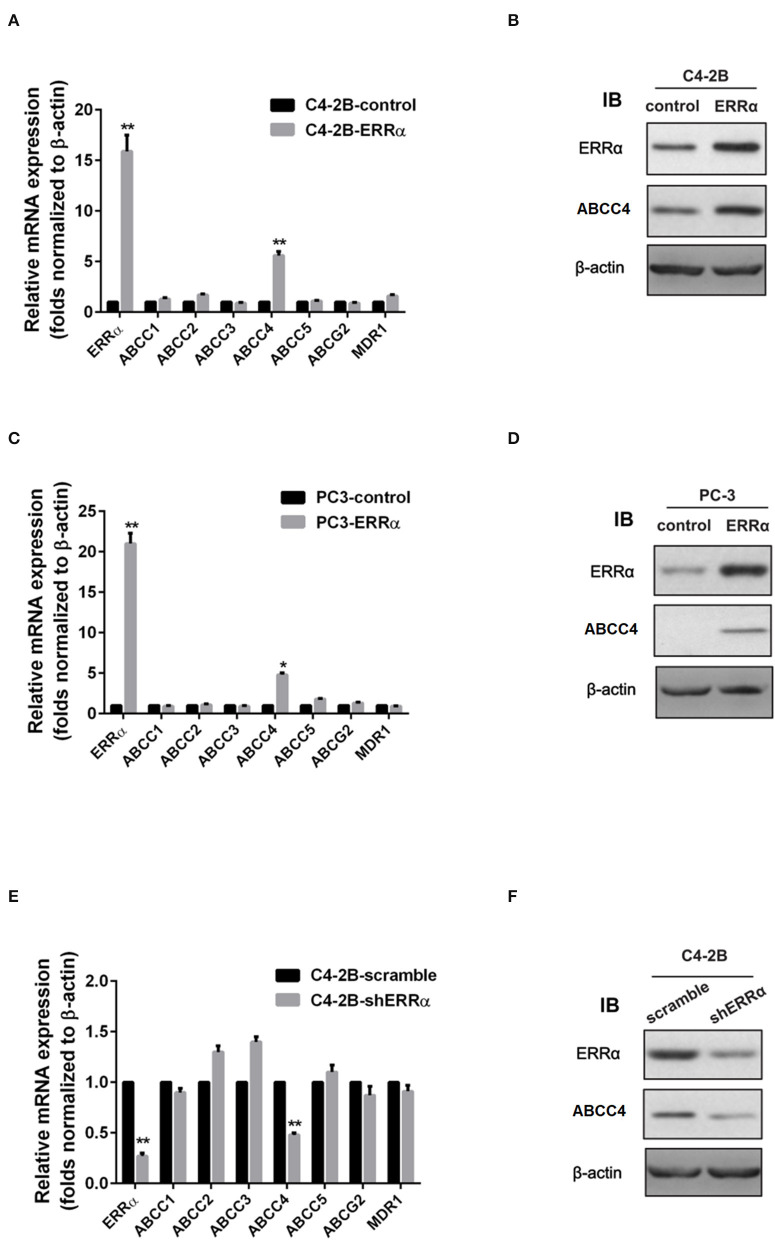
Regulation of ERRα on drug resistance-related genes. **(A,C)** mRNA expression of drug resistance-related genes after ERRα overexpression in C4-2B and PC3 cells. **(B,D)** Protein expression of ERRα and ABCC4 (MRP) after ERRα overexpression in C4-2B and PC3 cells. **(E)** mRNA expression of drug resistance-related genes after ERRα knockdown in the C4-2B cell. **(F)** Protein expression of ERRα and ABCC4 (MRP) after ERRα knockdown in the C4-2B cell. *p < 0.05, **p < 0.01 compared with respective control.

We then determined the expression of major drug resistance-related genes, including ABCC1-5, ABCG2, and MDR1, after ERRα manipulation. Overexpression of ERRα in both C4-2B and PC3 cells was accompanied by a significant upregulation of ABCC4 at the mRNA and protein levels (Figures 2A–D). Similarly, the knockdown of ERRα in C4-2B resulted in significant downregulation of ABCC4 (Figures 2E,F). These data indicated that ERRα may influence docetaxel sensitivity by regulating ABCC4 expression in PCa. Docetaxel works as a microtubule stabilizer, and overexpression of βIII-tubulin (TUBB3) was often found in docetaxel resistance in different cancers (32). Therefore, we also determined the relationship of ERRα and TUBB3 by bioinformatics analysis using TCGA prostate cancer data containing 551 samples (Supplementary Figure 2). Their expression showed some but not very strong positive correlation.

### Expression of ERRα and ABCC4 in Docetaxel-Resistant PCa Cells

**Figure 3:**
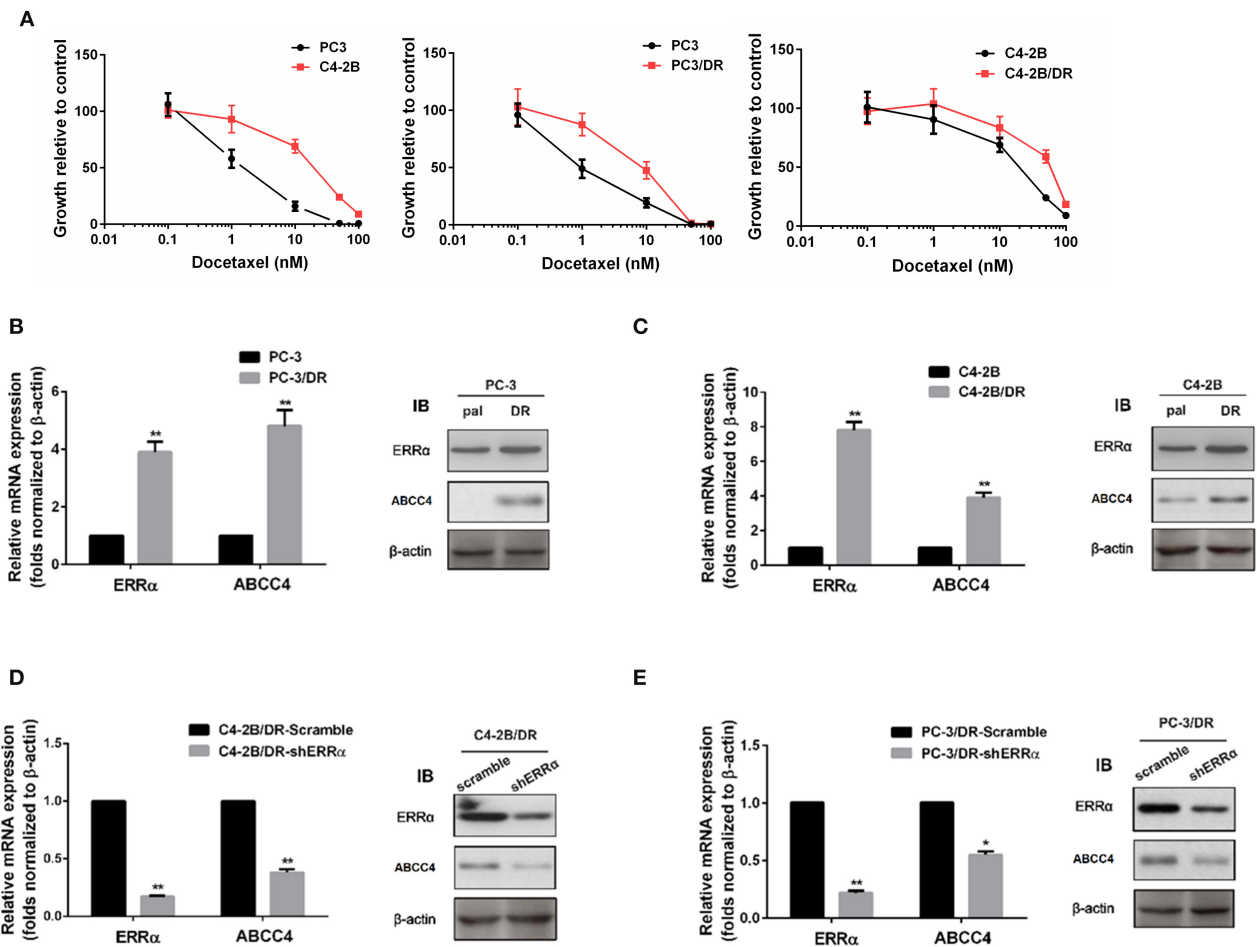
Expression of ERRα and ABCC4 in docetaxel-resistant cells. **(A)** Sensitivity of C4-2B, PC3, and their drug-resistant cells to docetaxel was determined by MTT assay. **(B,C)** Expression of ERRα and ABCC4 in docetaxel-resistant C4-2B and PC3 cells was increased compared with parental cells. **(D,E)** Expression of ERRα and ABCC4 was decreased after ERRα knockdown in drug-resistant C4-2B and PC3 cells. Pal, parental cell; DR, drug-resistant cell. *p < 0.05, **p < 0.01 compared with respective control.

To further elucidate the role of the ERRα and ABCC4 in docetaxel resistance, we determined the sensitivity of C4-2B and PC3 for docetaxel and established docetaxel-resistant C4-2B and PC3 cell lines (Figure 3A). PC3 was more sensitive to docetaxel than C4-2B. Consistent with our hypothesis that ERRα overexpression is involved in docetaxel resistance, higher expression of ERRα is found in docetaxel-resistant C4-2B and PC3 (Figures 3B,C). At the same time, ABCC4 was also highly expressed in docetaxel-resistant C4-2B and PC3 compared with their respective parental cells (Figures 3B,C). Moreover, the knockdown of ERRα by shRNA in the resistant cell lines reversed the high expression of ABCC4 (Figures 3D,E). Based on the above findings, we suspect that ERRα might exert a regulatory role on ABCC4.

### Intracellular Docetaxel Accumulation After ERRα Manipulation

**Figure 4:**
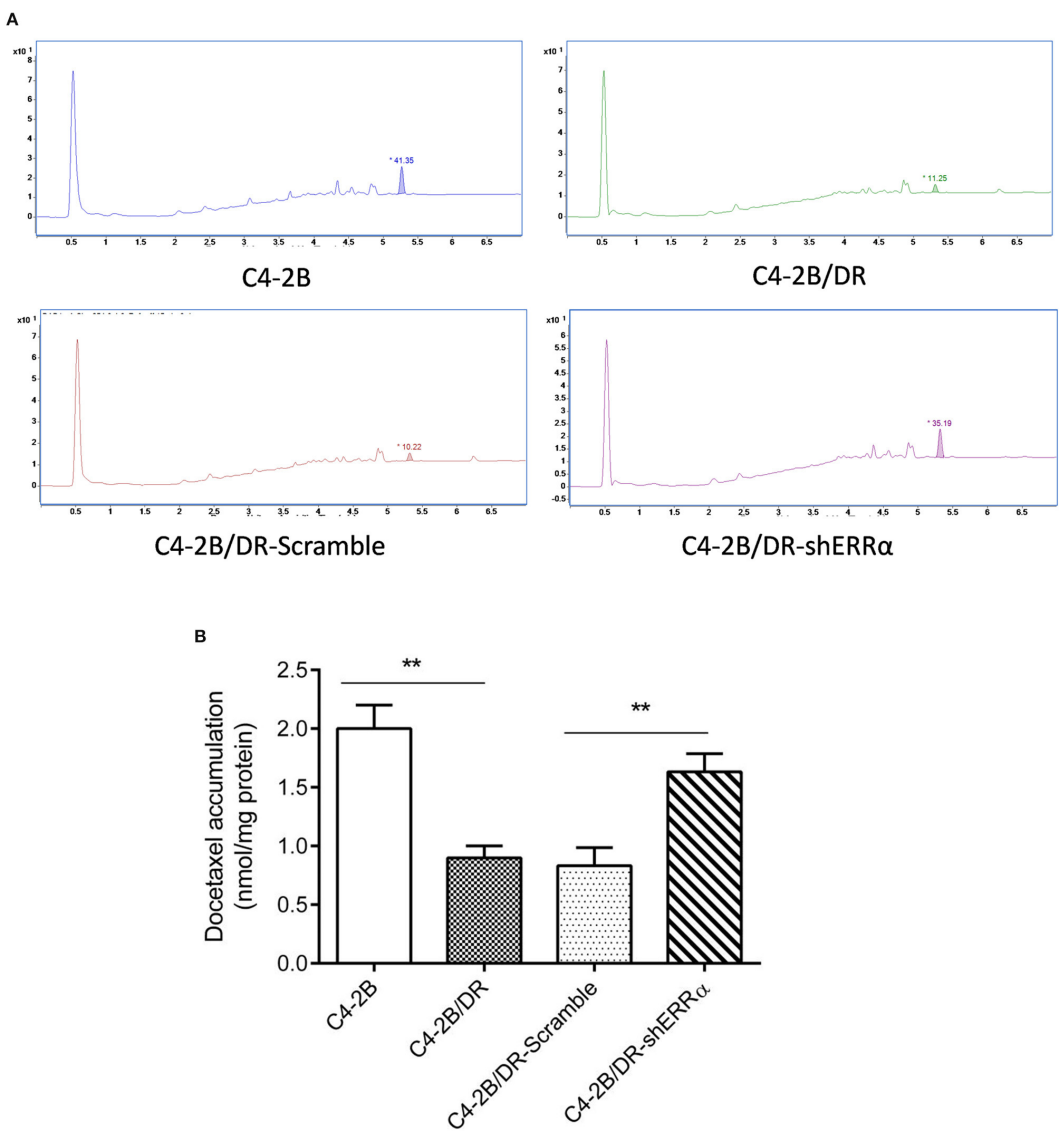
Knockdown of ERRα increased drug accumulation in docetaxel-resistant cell. **(A)** Representative HPLC figure of docetaxel accumulation in C4-2B and drug-resistant cells. **(B)** Statistical analysis of HPLC results. Intracellular docetaxel was significantly lowered in the drug-resistant C4-2B cell and the decrease was rescued by ERRα knockdown. **p < 0.01.

Since ABCC4 confers drug resistance through the efflux of anticancer drugs from cancer cells (33), we next determined whether intracellular docetaxel quantity was changed after ERRα manipulation. In drug-resistant C4-2B, there was a significantly lower intracellular docetaxel level due to the efflux of drugs (Figures 4A,B). Interestingly, after the knockdown of ERRα, the intracellular docetaxel level was significantly elevated, indicating that inhibition of ERRα could inhibit drug efflux associated with ABCC4.

### Interaction Between ERRα and ABCC4

**Figure 5:**
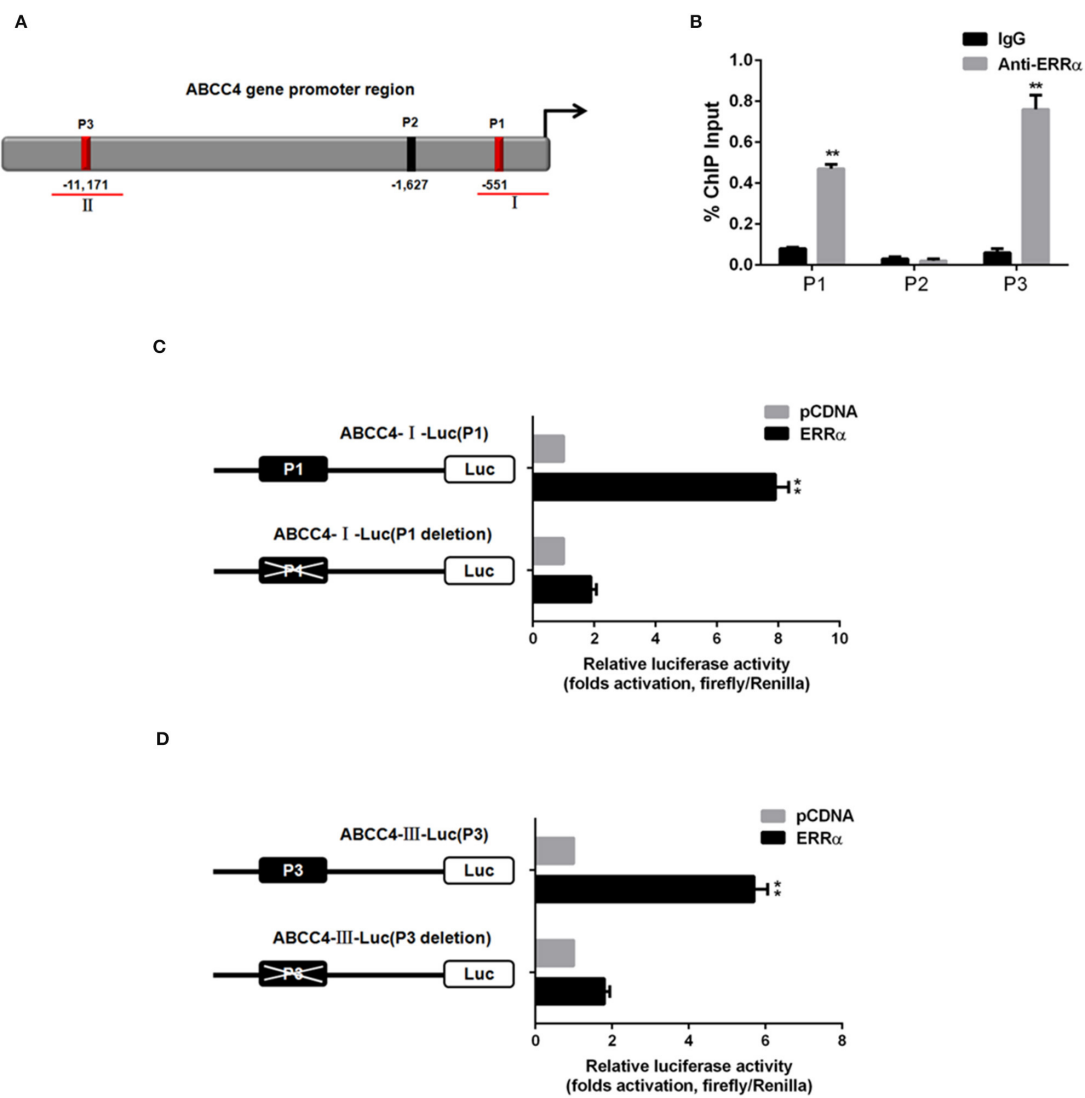
Direct interaction between ERRα and ABCC4. **(A)** Representative graph of ABCC4 promoter regions containing putative ERRα binding sites. **(B)** P1 and P3 regions were pulled down by ERRα antibody by CHIP assay. **(C)** Luciferase signal for plasmid-containing P1 region was increased by ERRα. **(D)** Luciferase signal for plasmid-containing P3 region was increased by ERRα. **p < 0.01 compared with respective control.

To verify whether ERRα directly regulates ABCC4, CHIP and luciferase assays were performed. Three potential ERRα binding sites (namely P1, P2, P3) were predicted in the ABCC4 gene promoter region (Figure 5A). The CHIP assay revealed that the ERRα occupancy was significantly elevated in the P1 and P3 regions on the ABCC4 promoter (Figure 5B). Subsequently, we constructed ABCC4 promoter luciferase reporters expressing promoter regions around P1 and P3 (ABCC4-I-Luc and ABCC4-III-Luc) and their respective mutant expressing the same region but with P1 or P3 deletion (Figures 5C,D). Results showed that after transfection with ERRα-overexpressing plasmids, both ABCC4-I-Luc and ABCC4-III-Luc exhibited enhanced luciferase signal, while their mutant counterparts did not show such enhancement (Figures 5C,D), indicating a direct binding of ERRα and the ABCC4 gene promoter regions P1 and P3. Taken together, our results demonstrated that ABCC4 is a direct target of ERRα.

### Bioinformatics Analysis and Validation

**Figure 6:**
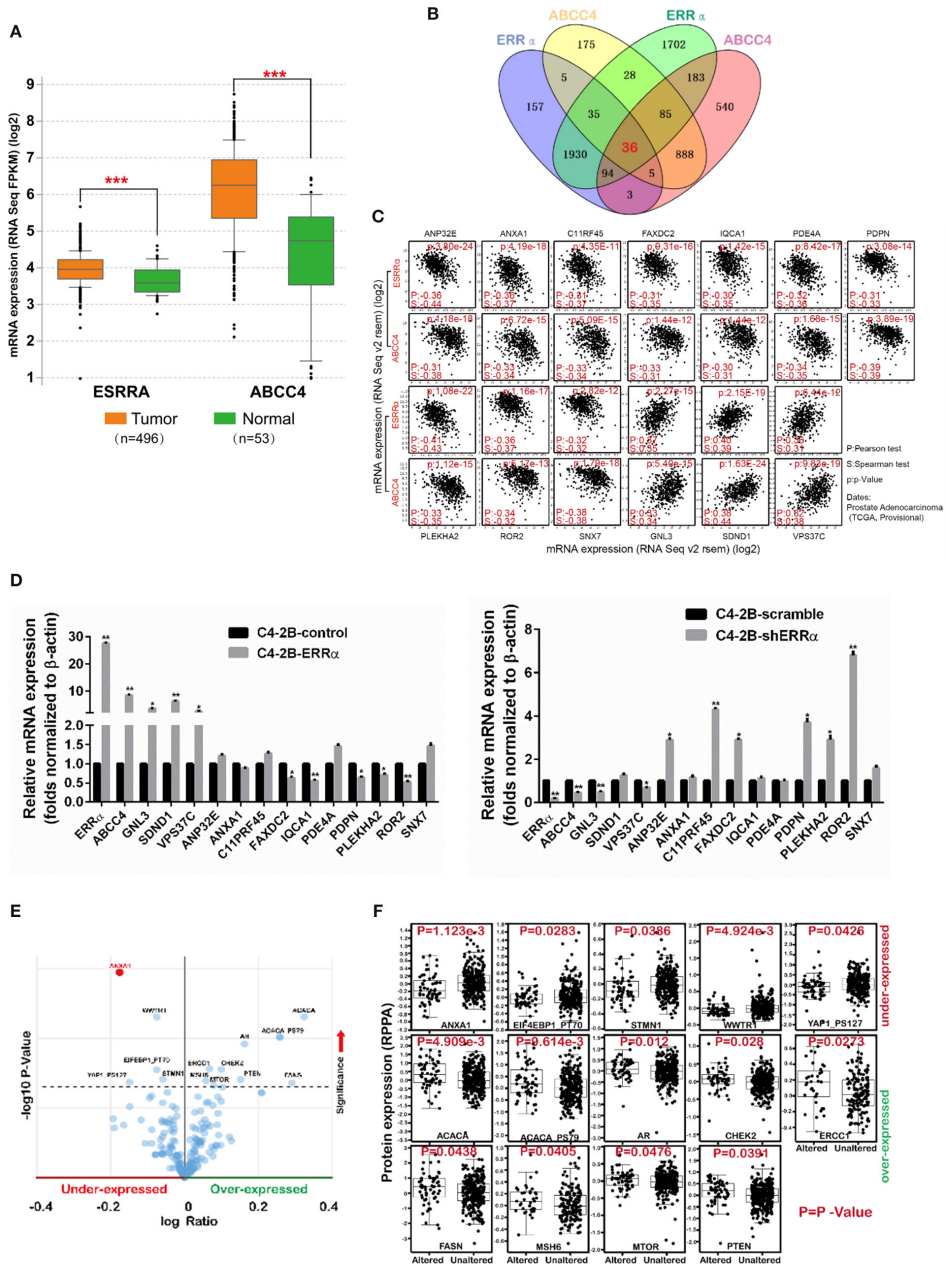
The expression level and potential targets of ERRα and ABCC4 from bioinformatics analysis. **(A)** The expression of ERRα and ABCC4 were elevated in prostate cancer using data from TCGA. **(B)** Genes co-expressed with ERRα and ABCC4 at mRNA level; 36 genes were co-expressed with both ERRα and ABCC4 from two independent data sets. Co-expression level was analyzed by Spearman's correlation test, and 0.3 cutoff was used. **(C)** Thirteen genes from **(B)** were significantly co-expressed with ERRα and ABCC4. **(D)** The 13 genes were validated by qPCR. **(E)** Genes with significant protein level change upon ERRα and ABCC4 gene alteration. **(F)** The expression level of 14 proteins was significantly affected by ERRα and ABCC4 gene alteration. *p < 0.01, **p < 0.001, ***p < 0.001 compared with normal sample.

We analyzed the expression level of ERRα and ABCC4 in cBioPortal. ERRα and ABCC4 were significantly upregulated in PCa in a data set consisting of 496 tumor vs. 53 normal samples (Figure 6A). To find out the potential targets of ERRα/ABCC4, we investigated genes co-expressed with ERRα and ABCC4 at mRNA level in cBioPortal using TCGA data consisting of two independent data sets (TCGA cell 2015, 333 samples and TCGA provisional, 499 samples downloaded on January 5, 2018). As shown in Figure 6B, 36 genes were co-expressed with ERRα and ABCC4 from both data sets. Among them, 13 genes showed a significant association with ERRα and ABCC4 (Figure 6C). Three genes (GNL3, SDND1, and VPS37C) were positively associated with ERRα and ABCC4 and others showed a negative correlation. The expression of these genes after ERRα manipulation was validated (Figure 6D). In accordance with the bioinformatics finding, GNL3 and VPS37C were positively correlated with ERRα and ABCC4, and FAXDC2, PDPN, PLEKHA2, and POP2 were negatively correlated with them.

We next analyzed proteins whose expressions were significantly affected by ERRα and ABCC4 gene alteration by protein enrichment analysis in cBioPortal. Results showed that 14 proteins were significantly influenced by ERRα and ABCC4 (Figures 6E,F), among which five (ANXA1, EIF4EBP1_PT70, WWTR1, STMN1, YAP1_PS127) were downregulated and accompanied by ERRα and ABCC4 alteration, and 9 (ACACA, ACACA PS79, AR, CHEK2, ERCC1, FASN, MSH6, MTOR, and PTEN) were upregulated (Figures 6E,F). Interestingly, the ANXA1 protein was decreased with ERRα and ABCC4 alteration (Figure 6F), and its mRNA was also negatively correlated with ERRα and ABCC4 (Figure 6C).

## Discussion

Docetaxel is effective against most of PCa; however, its resistance hampers the successful clinical use of docetaxel-based therapy. According to previous studies, docetaxel resistance has occurred in different kinds of PCa in clinic, such as hormone-refractory prostate cancer (HRPC), castration-resistant PCa, metastatic PCa, and so on. Recently, accumulating studies have focused on the mechanisms of docetaxel resistance in PCa. For example, ASC-J9, an AR degradation enhancer, combined with docetaxel can restore the docetaxel sensitivity and suppress castration-resistant PCa (34). ERRα, an orphan nuclear receptor, is implicated as an important regulator in drug resistance by many studies. It has been reported that in endometrial cancer, silencing ERRα can make HEC-1A cells more sensitive to paclitaxel and lead to a decreased tumor growth and angiogenesis (35). Pharmacological inhibition of ERRα could restore sensitivity to anti-cancer treatment in breast cancer (36, 37). In the current study, we sought to determine whether ERRα is involved in docetaxel resistance and whether targeting ERRα could overcome docetaxel resistance in PCa.

ERRα was first shown to be upregulated in prostate cancer in 2007, and its elevated expression was found to be associated with poor patient survival (17). It has been demonstrated that ERRα expression is correlated with castration-resistant prostate cancer, and it could promote tumor progression by targeting a number of cancer-related genes, including VEGF-A, WNT5A and TGFβ1, and WNT11 and HIF-1α (15, 38, 39). Recently, it has been shown that ERRα could promote the castration-resistant growth of PCa by regulating intratumoral androgen biosynthesis (40). However, the role of ERRα in the drug resistance of PCa is not well-elucidated. We found that overexpression of ERRα conferred tolerance to docetaxel and improved the survival of PCa cells (Figures 1A,B). Moreover, downregulation of ERRα sensitized a PCa cell to docetaxel treatment (Figure 1C), indicating ERRα was involved in docetaxel resistance. To elucidate the target of ERRα in regulating docetaxel resistance, we determined the impact of ERRα on major drug resistance-related genes. As shown in Figure 2, overexpression of ERRα led to a significantly elevated level of ABCC4, and the knockdown of ERRα was accompanied by a decrease in ABCC4 level, suggesting that ERRα contributed to docetaxel resistance probably through ABCC4. It has been reported that ABCC4 mRNA/protein expression was upregulated, and it was an important determinant of docetaxel resistance in PCa (17, 27, 41, 42). Recently, the inhibition of ABCC4 is confirmed as a potential cure for neuroblastoma by inhibiting tumor proliferation and sensitizing to chemotherapeutic drug (18). Notably, increased ABCC4 expression has a strong correlation with multidrug resistance in PCa (28, 43). Docetaxel works as a microtubule stabilizer, and it has been reported that docetaxel resistance was also related to the overexpression of βIII-tubulin (TUBB3) (32). Our bioinformatics analysis showed that the expression of ERRα and TUBB3 exhibited some positive correlation (Supplementary Figure 1). Whether ERRα could regulate TUBB3 expression in PCa warrants further study.

The involvement of ERRα in drug resistance has been widely studied in breast cancer and osteosarcoma (44–48). However, its role in PCa drug resistance was rarely examined. In our study, we established docetaxel-resistant PCa cell lines and found that both ERRα and ABCC4 were highly expressed in drug-resistant cells compared with their parental cells (Figures 3B,C). The knockdown of ERRα in resistant cells was accompanied by a decrease in ABCC4 (Figures 3D,E), suggesting that ERRα and ABCC4 were involved in docetaxel resistance in PCa. The importance of ERRα in drug resistance was functionally tested by docetaxel accumulation inside the drug-resistant cells after ERRα manipulation (Figure 4). The docetaxel level was significantly lower in resistant cells due to drug efflux, which could be reversed by ERRα knockdown, suggesting the potential clinical benefit of inhibiting ERRα to treat docetaxel-resistant PCa (Figure 4). Subsequently, the CHIP and luciferase assays were performed to assess the direct regulation of ERRα on ABCC4. Results showed that ERRα could directly bind two regions in the ABCC4 promoter (Figure 5). This is the first report on the direct regulation of ERRα on ABCC4.

We further confirmed our finding using publicly available data from PCa patients and discovered that the expression level of both ERRα and ABCC4 was significantly higher in the prostate tumor vs. the normal samples (Figure 6A). Next, in order to explore other potential mechanisms, we tried to find out the potential targets of the ERRα/ABCC4 axis by bioinformatics analysis. We first looked at genes that are co-expressed with both ERRα and ABCC4 (Figures 6C,D). Thirteen genes were significantly co-expressed with ERRα and ABCC4 using data from two patient cohorts (Figure 6D). Among them, ANP32E (49), ANXA1(50), PDE4A (51), PDPN (52), and ROR2 (53) play an oncogenic role according to the literature, which is contrary to their negative correlation with ERRα and ABCC4. Therefore, they might not be a direct target of ERRα/ABCC4, and their function is irrelevant to their co-expression with ERRα/ABCC4. GNL3 was one of the genes that were positively associated with ERRα and ABCC4. Earlier studies have reported that the elevated expression of GNL3 is related to cancer proliferation and metastasis (54). Recently, GNL3 is also considered as a susceptibility gene for PCa metastasis due to its expression involved in aggressive human PCa multiplication, migration, and invasion (55). Moreover, its positive correlation with ERRα and ABCC4 was validated by qPCR (Figure 6D). Thus, GNL3 can be considered as a potential target for ERRα/ABCC4 and awaits further validation.

Moreover, we analyzed protein expression of potential ERRα/ABCC4 targets (Figures 6E,F). In total, 14 proteins were significantly affected by ERRα/ABCC4 mutation. Of these, five were significantly decreased with ERRα/ABCC4 alteration, and nine were increased with ERRα/ABCC4 alteration (Figure 6F). Consistent with previous results that ANXA1 mRNA is negatively correlated with ERRα/ABCC4, its protein is decreased accompanying ERRα/ABCC4 alteration. Among other decreased proteins, STMN1 has been reported as an oncogene (56). Interestingly, WWTR1 and YAP1_PS127 both belong to the Hippo signaling pathway, and they are frequently hyperactivated in cancer (57). For the proteins that increased with ERRα/ABCC4 alteration, AR (androgen receptor) is expressed in primary and metastatic PCa, and downregulation of AR is considered a potential therapy for PCa (58, 59). CHEK2 is a gene involved in DNA repair and its mutation is associated with PCa (60). ERCC1 has been demonstrated to be an independent prognostic marker in PCa and a therapeutic target to sensitize cancer cells to chemotherapy (61, 62). FASN (fatty acid synthase), the key enzyme in the control of fatty acid synthesis, has received considerable attention as a therapeutic target in cancer including PCa (63, 64). MSH6, one of DNA mismatch repair genes, is overexpressed in PCa and is linked to genetic instability and tumor aggressiveness (65). mTOR belongs to the PI3K-AKT-mTOR signaling pathway, which is strongly involved in many cancers. It interplays with androgen receptor (AR) in PCa (66) and thus represents a potential target for PCa (67). PTEN is a well-known tumor suppressor and is also frequently lost in PCa (68). Taken together, many of the proteins that increased with ERRα/ABCC4 alterations are strongly involved in PCa and awaits further investigation, including AR, CHEK2, ERCC1, FASN, MSH6, and MTOR.

In summary, our findings reveal for the first time the involvement of ERRα in docetaxel resistance in PCa by directly regulating ABCC4. With the pharmacological inhibitors for ERRα available, it appears to be a promising potential adjuvant therapy for docetaxel-resistant PCa and warrants further investigation.

## Data Availability Statement

All datasets generated for this study are included in the article/Supplementary Material.

## Author Contributions

HH, JL, and JS conducted the experiments and drafted the manuscript. LL, XW, and YL collected and processed the data. YX, QZ, and ML analyzed and interpreted the data. PK and YZ prepared the figures. XL and SX revised the article critically for important intellectual content and approved the final version to be published. QW, WW, and ZX managed the project, designed the study, and provided the funding. All authors contributed to the article and approved the submitted version.

## Conflict of Interest

The authors declare that the research was conducted in the absence of any commercial or financial relationships that could be construed as a potential conflict of interest.

## Supplementary Material

Supplementary Figure 1 Colony formation assay in PC-3 cell after ERRα manipulation. ^**^*p* < 0.01, ^***^*p* < 0.001 compared with respective control.

Supplementary Figure 2 Correlation of ERRα and TUBB3 in prostate cancer. Data were extracted from TCGA (*n* = 551).
