# Supplementary figures and images for: Increased ABCC4 Expression Induced by ERRα Leads to Docetaxel Resistance via Efflux of Docetaxel in Prostate Cancer
Source: Front Oncol. 2020 Aug 28;10:1474. doi: 10.3389/fonc.2020.01474 (PMC7493678; doi:10.3389/fonc.2020.01474)

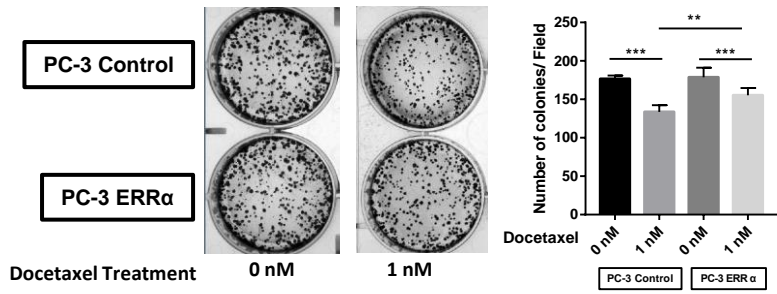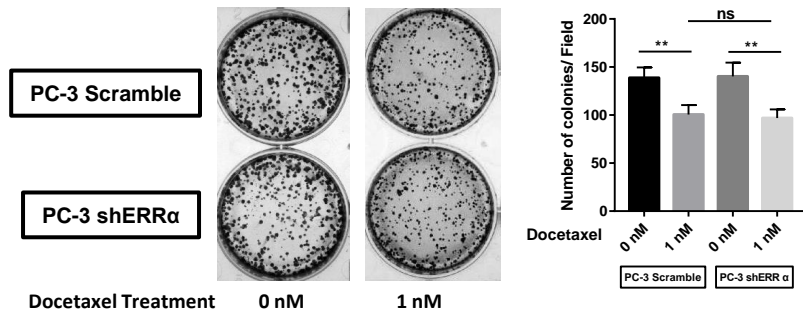

Supplement: Supplementary Figure 1 — Colony formation assay in PC-3 cell after ERRα manipulation. **p < 0.01, ***p < 0.001 compared with respective control. [file Image_1.pdf]

**Data 1**

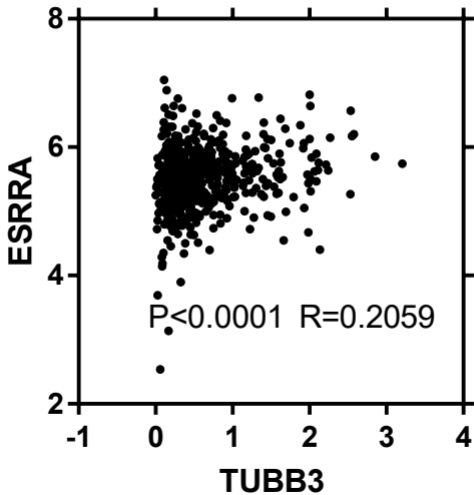

Supplement: Supplementary Figure 2 — Correlation of ERRα and TUBB3 in prostate cancer. Data were extracted from TCGA (n = 551). [file Image_2.pdf]
