# Supplementary material for: Increased ABCC4 Expression Induced by ERRα Leads to Docetaxel Resistance via Efflux of Docetaxel in Prostate Cancer
Source: Front Oncol. 2020 Aug 28;10:1474. doi: 10.3389/fonc.2020.01474 (PMC7493678; doi:10.3389/fonc.2020.01474)
Supplement: Supplementary file 3 [file Data_Sheet_1.docx]

**Colony formation**

Cells were inoculated in six-well plates at the density of 2000 cells/well. The culture medium was replaced every other day. After 14 days, the culture was terminated and the results were observed. After rising with PBS, cells were first fixed in 10% methanol for 30 min, and then stained in 5% crystal violet for another 30 min. Finally, the washing operation was repeated again and the number of clones was counted under a microscope (Olympus, Tokyo, Japan).
